# A randomized controlled trial of a new technique for laparoscopic management of ovarian endometriosis preventing recurrence and keeping ovarian reserve

**DOI:** 10.1186/s13048-019-0542-0

**Published:** 2019-07-20

**Authors:** Mohamed F. Shaltout, Ahmad elsheikhah, Ahmed M. Maged, Moutaz M. Elsherbini, Sherif S. Zaki, Sherif Dahab, Rasha O. Elkomy

**Affiliations:** Obstetrics and Gynecology Department, Cairo University, 481 King Faisal Street Haram, Giza, 12111 Egypt

**Keywords:** Ovarian endometrioma, Laparoscopic cystectomy, Drainage, Surgicel, Cyst recurrence, Ovarian reserve

## Abstract

**Introduction:**

Laparoscopic cystectomy provides more favourable outcomes as regards the recurrence and subsequent clinical pregnancy rates. It is associated with significant reduction in the ovarian reserve due to the inevitable removal of unaffected ovarian tissue. The aim of our study was to evaluate the efficiency of Surgicel in preventing recurrence of endometriomas after their laparoscopic conservative management (cystectomy or drainage).

**Material and methods:**

A randomized controlled trial included two hundred women (candidate for conservative laparoscopic management of ovarian endometriomas). They were randomized into four groups; group D in which patients underwent laparoscopic drainage of the endometrioma, group C in which patients underwent laparoscopic cystectomy of the endometrioma, group DS in which patients underwent laparoscopic drainage followed by insertion of Surgicel inside the cyst cavity & group CS in which patients underwent laparoscopic cystectomy of the endometrioma followed by insertion of Surgicel inside the remaining ovarian tissues. All patients were followed up for 2 years & the primary outcome was the recurrence of endometriomas in the ipsilateral ovary & the postoperative ovarian reserve was reassessed as a secondary outcome.

**Results:**

The Surgicel-treated groups had significantly lower hazard of recurrence compared to untreated groups (*p* = 0.004). Group CS had significantly lower hazard of recurrence compared to Group D & C (*p* = 0.014, 0.046 respectively). Group DS had significantly lower hazard of recurrence compared to Group D (*p* = 0.039) but it not significantly different from Group C (*p* = 0.112). Group DS had the lowest drop of AMH and was significantly lower than the other three groups.

**Conclusion:**

Surgicel reduces effectively the recurrence risk of endometriomas and its use during laparoscopic drainage is an effective alternative for traditional laparoscopic cystectomy with minimal affection of the patient ovarian reserve.

**Trial registration:**

Name of the registry: clinicaltrials.gov. Trial registration number NCT02947724. Date of registration October 28, 2016.

## Key message

Surgicel reduces effectively the recurrence risk with minimal affection of the ovarian reserve during laparoscopic management of ovarian endometriomas.

## Introduction

Endometriosis is a gynaecologic disease characterized by the presence of endometrial- like tissue outside the uterine cavity with the most common locations inside the pelvis being; the ovaries, the Douglas pouch & uterosacral ligaments [1]. It affects 6 to 10% of women in their reproductive age [2]. Classically, patients present with infertility, pelvic pain and/or a pelvic mass (endometriomas) [3]. Endometriomas are ectopic endometrium that grows within the ovarian tissue forming cystic structures filled with dark altered blood. They are detected in 17–44% of endometriosis cases [4].

Nowadays, ovarian endometriomas are managed by either cystectomy or drainage and ablation of the cyst wall. Based on Cochrane systematic review, laparoscopic cystectomy provides more favourable outcomes as regards the recurrence of endometriomas & subsequent clinical pregnancy rate when compared with drainage & ablation [5]. But unfortunately, two studies declared significant reduction in the ovarian reserve after surgical excision of endometrioma cyst wall due to the inevitable removal of unaffected ovarian tissue [6, 7]. More frustrating, recurrence rate is very high up to 30% [8]. Recurrence may be due to one or more of the following; de novo lesion, the regrowth of residual cells not removed during surgery or the growth of microscopic lesions unidentified at surgery [9].

The Surgicel® (oxidized regenerated cellulose - ORC) is a topical absorbable agent that has been introduced in surgical fields as an effective measure for haemostasis especially for oozing surfaces. In addition to the mechanical compression (tamponade –like) at the bleeding sites, Surgicel acts as a physical barrier that stimulates platelet aggregation and clotting. Furthermore, the acidic nature of ORC (pH ranges from 2 to 4) promotes haemostasis by triggering vasoconstriction & by the denaturation of blood proteins & the formation of artificial gel-like clot [10, 11]. ORC products are generally safe and well-tolerated as they rapidly cleared from implantation site, however, encapsulation of fluid and foreign body granulomatous reaction have been reported [11].

The existing scientific evidence considers laparoscopic cystectomy the surgical treatment of choice for endometriomas [4], however, recurrence is still a terrifying challenge. Elimination or postponing endometriomas’ recurrence is currently an unmet medical need that warrants further research.

In the current study we introduce and evaluate a new potential benefit for Surgicel in the laparoscopic treatment of endometriomas (cystectomy or drainage), namely its impact in reducing the rate of recurrence while preserving ovarian function.

## Material and methods

The present study was a prospective randomized controlled study conducted in Kasr El Aini hospital (faculty of medicine – Cairo University). Patients were recruited from gynaecology clinic then followed up in the period from October 2016 to January 2019. The study was approved by the Hospital Ethical Committee. All participants provided an informed written consent after explaining the aim of the study, the procedure & the potential hazards.

Women aged from 20 to 35 years and candidate for conservative laparoscopic treatment of ovarian endometriomas (either by drainage or cyst wall excision) were included. Participants were randomized into 4 groups; group D (drainage only) in which patients underwent laparoscopic fenestration and electrocautery of the endometrioma cyst wall, group C (cystectomy only) in which patients underwent laparoscopic excision of the endometrioma cyst wall, group DS (drainage & Surgicel) in which patients underwent laparoscopic fenestration of the endometrioma cyst wall followed by insertion of 4–8 pieces of Surgicel inside the cyst cavity, group CS (cystectomy & Surgicel) in which patients underwent laparoscopic excision of the endometrioma cyst wall followed by insertion of 4–8 pieces of Surgicel inside the remaining ovarian tissues. Randomization was done using computer generated random numbers.

Inclusion criteria included endometriosis-related clinical manifestations (infertility, pelvic pain or pelvic mass), unilateral & unilocular endometrioma (≥5 cm), rapidly growing endometrioma & good ovarian reserve (antimullerian hormone {AMH} > 1 ng/ml & antral follicular count {AFC} > 4). Recurrent & bilateral cases were excluded. In addition, patients who were unfit for surgery, suffered chronic diseases (e.g. cardiac disease or diabetes) or had any contraindication for laparoscopic surgery (excessive anterior abdominal wall scarring) were also excluded.

For all patients, full history was taken followed by complete physical examination & laboratory investigations investigations (AMH & routine preoperative investigations). Serum AMH was assayed by ELISA (enzyme linked immunosorbent assay) technique, using AMH Gen II ELISA kits (Expected Values: 0.9–9.5 ng/ml). Kits were purchased from Beckman Coulter, Inc., USA. Day 2 transvaginal ultrasound (TVUS) was done using a 7.5 MHz vaginal probe of the General Electric Voluson E8 ultrasound unit (GE Healthcare Austria GmbH, Seoul, Korea) to confirm the presence and assess the size and side of the endometrioma (ovarian cyst with homogeneous low-level ground glass echogenicity of the cystic fluid) & to assess the AFC (Number of visible follicles from 2 to 10 mm) in both the affected and healthy ovary.

Cystectomy or drainage was done by one of the investigators (FS). In cystectomy groups (C&CS), a small window (2 cm) was done in the cyst wall using diathermy or scissor followed by aspiration of the chocolate material from the cyst then stripping the cyst wall from ovarian tissue using 2 non-traumatic graspers (by traction-counter traction technique) and finally irrigating the remaining ovarian tissues with normal saline solution. In drainage groups (D & DS), a small window (1 cm) was done in the cyst wall using diathermy or scissor followed by aspiration of the chocolate material from the cyst & then irrigation of the cyst cavity with normal saline solution till complete elimination of the chocolate material. In non-Surgicel groups (D&C), haemostasis & ablation of the remaining endometriotic cyst wall was done by bipolar electrocautery. In Surgicel groups (DS&CS), each SURGICEL® (oxidized regenerated cellulose - Ethicon US, LLC.) knitted fabric (5 × 10 cm) was divided into four equal pieces. Four to eight dry Surgicel pieces (according to the size of endometrioma) are inserted inside the cavity of the cyst (group DS) or the remaining ovarian tissues (group CS) then the Surgicel pieces were irrigated by 10 ml normal saline solution. If the ovarian edges were gaped, approximation was done using 1–3 interrupted sutures of 4/0 polydioxanone (PDS® Suture - Ethicon US, LLC.). All patients were followed up every 3 months for 2 years following the laparoscopic surgery. No postoperative hormonal treatments were given after the surgical intervention for all participants during the follow-up period. The primary outcome was the recurrence of endometriomas in the ipsilateral ovary (recurrence was defined as the presence of ovarian cysts with the characteristic sonographic features of endometriomas (≥1 cm). The ovarian reserve was reassessed (AMH & day 2 AFC) as secondary outcome 6 months following the laparoscopy. All ultrasounds (for initial pre-operative assessment & post-operative follow-up) were done by single investigator (Ahmad elsheikhah).

### Statistical methods

Statistical analysis was done using IBM© SPSS© Statistics version 22 (IBM© Corp., Armonk, NY, USA). Numerical data were expressed as mean and standard deviation or median and range as appropriate. Qualitative data were expressed as frequency and percentage. Chi-square test (Fisher’s exact test) was used to examine the relation between qualitative variables. For normally distributed quantitative data, comparison between the 4 groups was done using ANOVA test, then post-Hoc “Schefe test” was used for pair-wise comparison. The percentage of change between pre- and post-procedural readings of AMH and AFC was calculated and compared using Kruskal-Wallis test due to non-normal distribution followed by pairwise comparison. Survival analysis was done using Kaplan-Meier method to calculate the hazard of recurrence in the four groups that was compared using log-rank test. Cox proportional hazard was used to estimate the hazard ratio (HR) of recurrence in the studied groups using Group D as the reference group. HR was expressed with its 95% confidence interval (CI). All tests were two-tailed. A *p*-value < 0.05 was considered significant.

### Sample size calculation

There is no previous study investigating the effect of Surgicel application in cases of ovarian endometriosis. We assumed that a 20% reduction of the risk of recurrence will be considered clinically meaningful. Based on this, a total sample of 192 patients will be required to elicit the treatment effect at an alpha level of **0.008** (corrected with Bonferroni correction for multiple comparisons) and a power of the study of 80%. Considering the probability of dropouts during follow up, the number of cases was further raised to 200 patients. The sample size was estimated using the G*Power© software (Institutfür Experimentelle Psychologie, Heinrich Heine Universität, Düsseldorf, Germany) version 3.1.9.2.

## Results

**Fig. 1:**
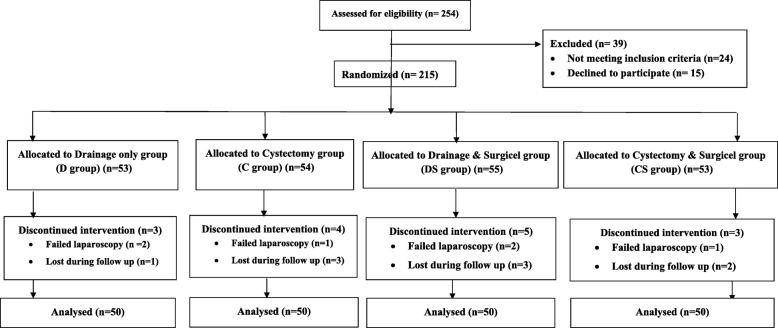
Consort flow of patients through the study

**Fig. 2:**
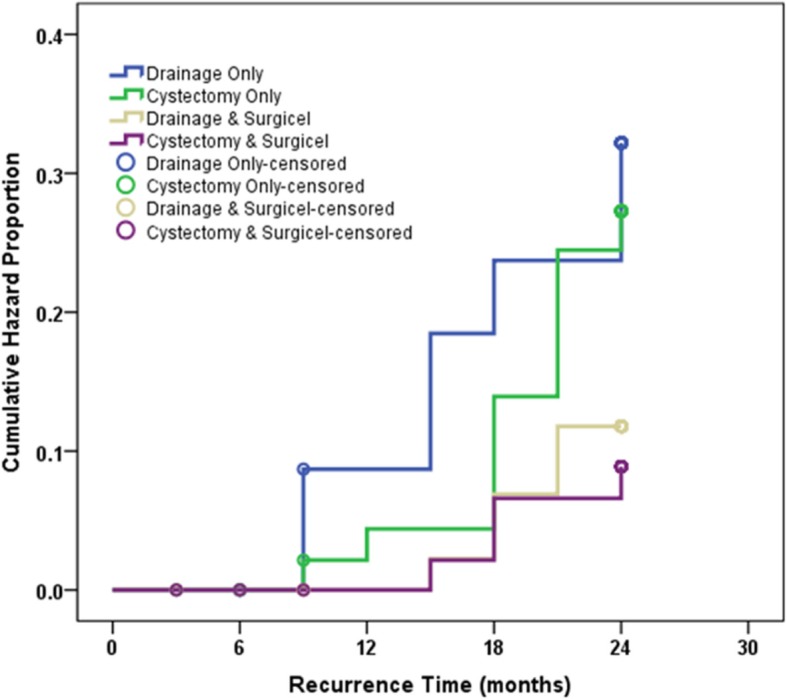
Cumulative hazard of recurrence at 24 months of the four studied groups

**Fig. 3:**
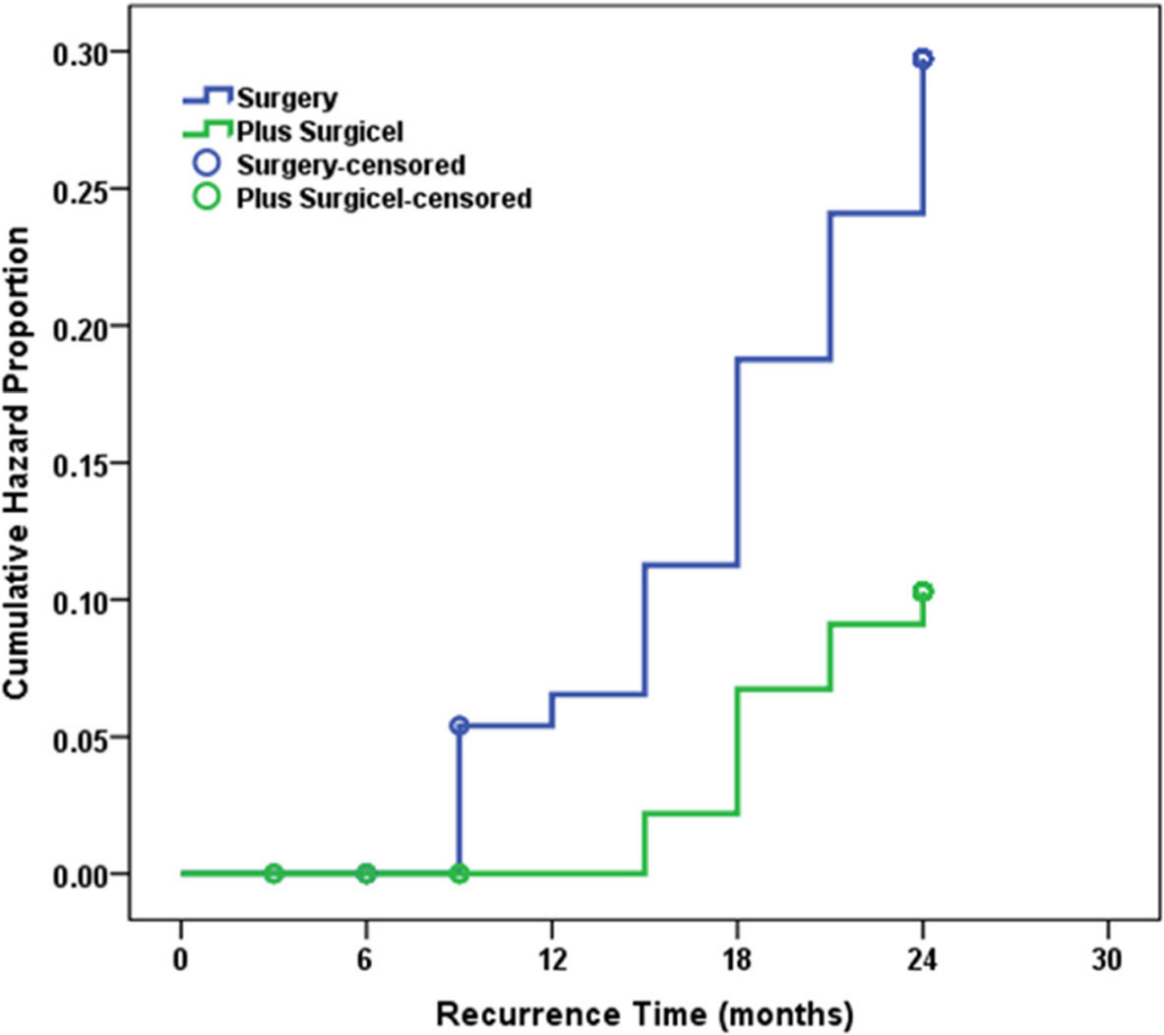
Cumulative hazard of recurrence at 24 months of Surgicel-treated and untreated patients

Overall, 215 patients underwent randomization. The flow of patients in the current study were summarized in Fig. 1. The four groups were comparable regarding the basic characteristics including age, BMI, type of presentation and size and side of the lesion (Table 1). The Surgicel-treated groups had significantly lower hazard of recurrence compared to untreated groups (*p* = 0.004, Figs. 2 & 3). Meanwhile the two Surgicel-treated groups were comparable (*p* = 0.680) and likewise the two untreated groups (*p* = 0.605). Surgicel-treated patients underwent laparoscopic cystectomy (Group CS) had significantly lower hazard of recurrence compared to those underwent drainage (Group D; *p* = 0.014) or cystectomy (group C; *p* = 0.046) while patients in “drainage and Surgicel” group (Group DS) had significantly lower hazard of recurrence when only compared to those in “drainage” group (*p* = 0.039) (Tables 2 & 3). The impact on ovarian reserve is summarized in Table 4. The four groups showed decrease of AMH levels after treatment (Group DS had significantly the lowest drop of AMH compared to three groups) and the AFC in the operated ovary showed significantly higher decrease in Group D compared to Group CS (*p* = 0.021). A total of 17 women got pregnant spontaneously by the end of follow up period; 10 in Surgicel treated groups (6 in cystectomy and 4 in drainage) and 7 in the non-surgicel group (2 in drainage and 5 in cystectomy). Using survival analysis, there was no significant difference between the two groups in pregnancy rate at 24 months (*p* = 0.543). No eventful complications or side-effects (in the form of allergic or foreign-body reactions, infection, abscess and granuloma formation) were recorded in the Surgicel groups.

**Table 1:** Baseline demographic and clinical characteristics of the four studied groups

	Drainage Only (D) *n* = 50	Cystectomy Only (C) *n* = 50	Drainage & Surgicel (DS) *n* = 50	Cystectomy & Surgicel (CS) *n* = 50	*p* value
Age (years)	28.2 ± 4.1	26.6 ± 4.4	27.5 ± 3.7	27.9 ± 4.1	0.210
Body mass index (kg/m^2^)	25.5 ± 1.3	25.3 ± 1.4	25.4 ± 1.3	25.3 ± 1.2	0.884
Presentation					0.824
Primary Infertility	16 (32%)	20 (40%)	15 (30%)	15 (30%)	
Secondary infertility	16 (32%)	12 (24%)	18 (36%)	18 (36%)	
Pelvic Pain	12 (24%)	11 (22%)	7 (14%)	9 (18%)	
Pelvic Mass	6 (12%)	7 (14%)	10 (20%)	8 (16%)	
Size of the lesion (cm)	6.4 ± 1.1	6.3 ± 1.1	6.5 ± 1.1	6.5 ± 1.2	0.791
Side of the lesion					1.000
Right	26 (52%)	25 (50%)	26 (52%)	26 (52%)	
Left	24 (48%)	25 (50%)	24 (48%)	24 (48%)	

Data expressed as mean ± SD or No. (%)

**Table 2:** Cumulative hazard of recurrence at 24 months of the four studied groups

Group	No. of Cumulative Events	Cumulative Hazard Proportion at 24 months	*p* value
Drainage Only (D)	13/48	27.1%	0.031
Cystectomy Only (C)	11/45	24.4%	
Drainage & Surgicel (DS)	5/46	10.9%	
Cystectomy & Surgicel (CS)	4/44	9.1%

**Table 3:** Hazard ratio for different procedures versus drainage only using Cox-proportional hazard method

Procedure	*p* value	Hazard Ratio	95% CI
Cystectomy only	0.598	0.806	0.361–1.799
Drainage + Surgicel	0.049	0.355	0.126–0.995
Cystectomy + Surgicel	0.022	0.271	0.088–0.831

*CI* Confidence interval

**Table 4:** Percentage change of antimullerian hormone level and antral follicle counts after treatment in the four studied groups

	Drainage Only (D) *n* = 50	Cystectomy Only (C) *n* = 50	Drainage & Surgicel (DS) *n* = 50	Cystectomy & Surgicel (CS) *n* = 50	*p* value
AMH					
Median	− 33.5^a^	−54.1^b^	−17.3^c^	−45.4^ab^	< 0.001
Range	−65.3 to −23.6	− 75.8 to − 36.1	− 23.8 to − 9.8	−79.7 to − 28.7	
AFC (S)					
Median	0.0^a^	12.5^ab^	0.0^ab^	0.0^b^	
Range	−100.0 to 100.0	−100.0 to 100.0	−50.0 to 200.0	−50.0 to 200.0	0.013
AFC (C)					
Median	0.0	−11.1	0.0	−12.5	
Range	−33.3 to 25.0	−33.3 to 33.3	−22.2 to 50.0	−28.6 to 40.0	0.061
AFC (T)					
Median	−9.1^a^	0.0^ab^	0.0^b^	0.0^ab^	
Range	−33.3 to 33.3	−37.5 to 28.6	−15.4 to 33.3	−20.0 to 33.3	0.007

*AMH* Antimullerian hormone, *AFC* Antral follicle count, (*S*) Same side, (*C*) Contralateral side, (*T*) Total

Groups with different superscript letters are significantly different

## Discussion

Endometriomas are adnexal masses commonly encountered in patient suffered from endometriosis. They either impair fertility or induce pelvic pain. Three main theories are claimed to be responsible for their pathogenesis (celomic metaplasia of ovarian inclusion cyst, endometriotic transformation of functional cysts or endometrial implant bleeding). Management plan for endometriomas is a controversial matter and depends on various issues including symptoms, woman age, desire of fertility, risk of malignancy, pre-management ovarian reserve, previously used treatment lines & cyst features (i.e., size, laterality & location). Many conservative procedures are described including aspiration (ultrasound guided or laparoscopic), drainage and ablation of the remaining cyst wall by electrocautery or laser & cystectomy [4, 12].

In all procedures, recurrence remains a challenge to the surgeons who must balance complete eradication of the endometriotic tissue against inadvertent destruction of healthy ovarian tissue and compromising ovarian reserve. It is when facing that dilemma that we stumbled upon the therapeutic benefit of Surgicel in treatment of endometrioma. Initially used to cover the cyst bed to control the bleeding, we noticed on patient follow up the low incidence of endometrioma recurrence & the current study proved this finding.

Ultrasound-guided aspiration of endometrioma carries a very high risk of recurrence (up to 90% within 1 month). Consequencely, it was not widely used and proposed as an alternative therapeutic procedure in certain patients (e.g., for the relief of pelvic pain or to improve reproductive outcome in infertility patients) [13].

Endometrioma drainage followed by ablation of the remaining cyst wall is an alternative procedure that significantly improve pelvic pain but recurrence still a major risk. Laparoscopic cystectomy is the first choice for conservative management of endometrioma [12, 14]. It carries the following benefits; decreases recurrence rates (it ranges from 9.6 to 45% after one surgery), increases spontaneous pregnancy rate (14–54%) & reduces pelvic pain [15–17]. However, the main problem with this technique is the destruction & removal of healthy ovarian stroma resulting in decreasing the ovarian reserve postoperatively. Moreover, ovarian failure was reported after bilateral procedure [12, 18]. Additionally, laparoscopic cystectomy often proves difficult as the cyst wall is tightly adherent to ovarian tissue. This leads in times to incomplete removal of the cyst and consequently recurrence. Following cystectomy, the bleeding bed is either cauterized or the ovary sutured using intracorporeal sutures. Cauterization may prove detrimental to the ovarian reserve, as well as causing adhesions.

Post-operative hormonal modalities had been used in the treatment of endometriomas with doubtful benefits. Based on Brown’s Cochrane review, there was no evidence of benefit with post-operative medical treatment for endometriomas & there was no evidence that hormonal treatment improved clinical pregnancy rates [5].

This effect of endometrioma removal on ovarian reserve has been evaluated using a variety of tests including serum AMH measurement, AFC and the number of recruited follicles in response to ovarian stimulation. Raffi and his colleagues in their meta-analysis studied the impact of endometrioma cystectomy on AMH level. They reported that cystectomy significantly reduced AMH levels postoperatively (WMD: -1.13; 95% CI: − 0.36 to − 1.88). likewise, Urman and his co-workers (2013) evaluated the effect of unilateral endometrioma cystectomy on serum AMH (pre, immediate and remote postoperative) and AFC. AMH and AFC showed immediate (24 and 11%, respectively) and sustained (24 and 15%, respectively) reduction after surgery and the reduction was not correlated with the use of bipolar electrocautery during surgery [7, 19]. On the other hand, several studies reported that serum AMH changes are dependent on the excised endometrioma characteristics. Wang and his co-workers reported significant long-time AMH decrease in patients with larger, bilateral cysts and in stage IV endometriosis compared to short-time decrease in smaller, unilateral cysts and stage III. Even more, lower drop in postoperative AMH when using suturing technique instead of coagulation [20, 21].

The present study demonstrated that the use of Surgicel during laparoscopic cystectomy or drainage of endometriomas causes further reduction of their recurrence (group CS had the lowest hazard of recurrence among the four groups). Even more, in cases managed by drainage, filling the remaining cavity with Surgicel® (group DS) reduces recurrence risk with overall results better than drainage only (group D) & comparable to traditional cystectomy (group C and group CS). Furthermore, in the DS group, avoiding electrocautery (depending on the haemostatic properties of Surgicel) seems to have preserved patient ovarian reserve to the maximum (group DS had the lowest drop in AMH) which make this procedure good alternative to cystectomy & excellent choice for patients with poor ovarian reserve. It postulates that ORC exerts a form of chemical ablation to the ectopic endometrium glandular tissue at a cellular level. This effect can be explained by the same mechanism of hemostasis exerted by ORC. Surgicel induces highly acidic environment (pH 2–4) & trigger severe vasoconstriction (resulting in tissue anoxia) within the treated endometrioma which may results in death of the remaining endometrial cells [10, 11].

The use of Surgicel (either during cystectomy or drainage of endometriomas) was generally safe with no eventful complications or side-effects recorded. This was in accordance with several studies that declared the safety of ORC being sterile and bioabsorbable products. Remote complications (e.g., chronic inflammation, infection, and foreign-body granulomatous formation) had been reported [11, 20]. However, the evidence-based data on the Surgicel optimal use and potential hazards is still lacking.

From a practical point of view, treatment cost must be taken into consideration. Use of Surgicel® is cheaper than equipment used for electrocauterization. It does not require the training and dexterity need to perform intracorporeal suturing and shortening of operative time is a further bonus.

The main limitations in the current study are that the pelvic pain improvement & patient satisfaction rate were not in our scope during the follow up period in addition to the small sample size & the lack of evidence about long-term recurrence and ovarian performance (due to the short duration of follow-up). Further large well-designed long-term studies are warranted before complete establishment of this technique. The study population included patients suffered endometriomas and the statistical analysis did not focus on the infertile patients per say. We focused on the impact of using the Surgicel on the ovarian reserve in both fertile and infertile patients. In future study, similar methodology may be applied on infertile patients with endometriomas.

In conclusion, the present study has demonstrated that Surgicel reduces effectively the recurrence risk of endometriomas following either laparoscopic cystectomy or drainage. Furthermore, laparoscopic drainage followed by filling the remaining cyst cavity with Surgicel is an effective alternative for traditional cystectomy that minimally impairs the patient ovarian reserve.

## Data Availability

Not applicable
